# Composition-Dependent Creep Resistance and Strain Rate Sensitivity of BCC Mg-Sc Alloy Studied via Nano-Indentation on Diffusion Couple

**DOI:** 10.3390/ma18163828

**Published:** 2025-08-15

**Authors:** Chenyue Liu, Guanglong Xu, Fuwen Chen

**Affiliations:** College of Materials Science and Engineering, Nanjing Tech University, Nanjing 211816, China

**Keywords:** Mg-Sc BCC alloy, diffusion couple, nano-indentation, creep resistance, strain rate sensitivity

## Abstract

Mg-Sc body-centered cubic (BCC) phase-structured alloys not only exhibit superior room-temperature ductility and quasi-isotropic deformation behaviors compared to conventional hexagonal close-packed (HCP) Mg alloys in mechanical applications, but they also demonstrate a shape-memory effect that is applicable to intelligent devices. Due to the introduction of a dual-phase microstructure feature, the unveiled strengthening/toughening mechanism, and the potential benefit of Sc alloying in BCC creep deformation, it is necessary to investigate the composition and time-dependent creep behaviors of BCC Mg-Sc alloys, such as creep resistance and strain rate sensitivity at room temperature, through nano-indentation on the Mg-Sc diffusion couple. A critical finding is that as the Sc content increases from 23.01 at.% to 33.56 at.%, the BCC Mg-Sc alloy exhibits a progressive enhancement in creep resistance at room temperature, evidenced by the creep stress exponent (*n*) rising from 49.02 to 66.22. Furthermore, the strain rate sensitivity (*m*) increases from 0.02 at 26.94 at.% Sc to 0.11 at 32.63 at.% Sc, along with the Sc composition gradient. These phenomena can be attributed to the formation of ordered structures with the increasing Sc concentration, which introduce short-range local barriers to dislocation motion, as confirmed through atomic-scale microstructural analysis.

## 1. Introduction

Magnesium alloys have received significant interest in the field of engineering due to their good castability [1], high specific stiffness [2,3], and superior electrical conductivity [4,5], with wide applications in the 3C and transportation sectors [6,7,8]. Rare-earth elements, such as Gd [9], Y [10], Nd [11], etc., are commonly employed for alloying the hexagonal close-packed (HCP) lattice of Mg and introducing thermally stable precipitates [10,12] to enhance heat resistance and creep resistance [9,10,11]. Nevertheless, HCP-structured Mg has an intrinsic problem of inadequate plasticity deformation [13].

Recent years have witnessed a growing interest in body-centered cubic (BCC)-structured Mg alloys. Mg-Li BCC alloys have good performance in terms of ductility but suffer from inherently low creep resistance [14]. Apart from Li, only Sc can be solid-solutioned with Mg atoms and shaped into a BCC solid solution with a broad composition across a varying range [15]. Mg-Sc alloys have been found to be functional materials featuring a shape-memory effect, strain glass, and biocompatibility [16,17,18,19]. Meanwhile, as structural materials, they open up a novel pathway of dual-phase strengthening, similar to Ti alloys, beyond dispersed precipitation hardening [20,21,22,23,24]. Mg-Sc BCC matrix alloys are as light as organic-based materials with a density of ~2 g· cm^–3^, possess a nearly temperature-independent and low Young’s modulus of ~20–23 GPa from room temperature down to ~100 K [18], a Vickers hardness of 231.5 Hv [22], a yield strength of ~200–270 MPa [19], an ultimate tensile strength of 310 MPa, an elongation of 28.8% [25], and a long fatigue life, and can overcome the tradeoff between the strength and ductility of conventional Mg alloys [19].

In addition, Sc lies in the IIIB group of the periodic table, the same as rare-earth elements. The effect of Sc on creep resistance has been studied for HCP-structured Mg alloys [26,27,28,29,30]. The improvement in creep resistance by Sc alloying is associated with the pinning of Sc-containing short-range particles, which influences the cross-slip of dislocations [31,32,33], and a low tendency for Sc diffusion, which imposes drag on migrating grain boundaries [34]. However, the effect of Sc alloying on the creep resistance of BCC-structured Mg alloys remains limited. In particular, creep displacement, the creep stress exponent, and strain rate sensitivity are critical parameters for evaluating creep deformation behaviors [35]. As such, a systematic investigation into the composition dependence of creep resistance-relevant parameters for Mg-Sc BCC alloys is necessary.

The employment of a conventional uniaxial tensile creep test to study creep deformation behaviors at the macro-scale is time-consuming and cost-intensive [36]. This has advanced the adoption of nano-indentation as a novel approach to investigate creep deformation behaviors at the micro-scale, enabling the evaluation of both time-dependent creep deformation and strain rate sensitivity [37], and this technique has been successfully implemented to study these phenomena in pure Mg and Mg-X (X = Al, Ca) systems [38,39,40]. Recent advances include the combination of nano-indentation with a solid-state diffusion couple [41,42] (which has the ability to introduce a continuous composition gradient) to serve as a high-throughput method to screen for composition-dependent mechanical properties [43].

Building upon these technical developments, the present work employs a synergy of the solid-state diffusion couple and nano-indentation to address the lack of data on composition-dependent creep behavior in BCC Mg-Sc systems at room temperature. The variations in the creep displacement, creep stress exponent (*n*), and strain rate sensitivity (*m*) with Sc contents are evaluated. It is noteworthy that this work focuses on the intrinsic creep resistance and the intrinsic strain rate sensitivity purely as influenced by the chemical composition of Sc at room temperature. Other effects of thermal-activated deformation [44], such as grain boundary sliding and grain boundary diffusion at elevated temperatures, are not involved.

## 2. Materials and Methods

### 2.1. Fabrication of Diffusion Couple

**Figure 1:**
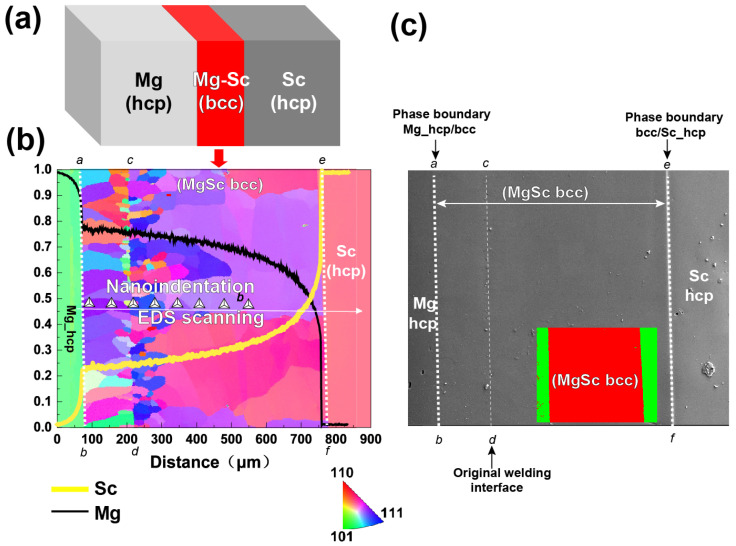
(**a**) Schematic drawing of sandwich three-phase structure in the diffusion couple. The BCC phase in red was generated in annealing. (**b**) EBSD orientation map of diffusion couple superposed with EDS component–distance curves (yellow curve is for Sc composition and black curve is for Mg composition), and schematic drawing of nano-indentation positions showing using triangles (not experimental observation). (**c**) Back-scattered SEM image and phase identification map. The phase boundaries and initial wielding interface are also highlighted.

A Mg-Sc diffusion couple (see the schematic drawing in Figure 1) was synthesized through solid-state diffusion bonding. The end members were pure Mg (99.95 wt.%) and Sc (99.9 wt.%) blocks (Jinyu Sunshine Metallic Materials Co., Ltd., Beijing, China), which were homogenized at 450 °C (723 K) and 800 °C (1073 K), respectively, for 48 h under an argon atmosphere. Rectangular specimens with the size of 10 mm × 10 mm × 5 mm were sectioned from homogenized ingots using wire electrical discharge machining, followed by mechanical polishing to achieve mirror-like surfaces. The polished Mg and Sc blocks were mechanically clamped in a stainless-steel fixture to ensure intimate interfacial contact. The assembled diffusion couple was sealed within an evacuated quartz tube, backfilled with high-purity Ar. It was then subjected to annealing at 630 °C (903 K) for 72 h, also in an Ar atmosphere, to form an intermediate BCC phase, the diffusional interfaces of Mg-rich HCP/BCC and BCC/Sc-rich HCP, as well as the chemical composition gradients in individual phases. The annealing temperature and time were set based on the previous work of the present authors [25,45,46], ensuring a thickness of the generated BCC layer of more than 1mm, a sigmoid shape of composition–distance profiles, and an appropriate BCC grain size distribution. Annealing in Ar prevented oxidation and other contamination, minimized the volatilization of Mg via Le Chatelier’s principle, and ensured microstructural stability. Post-diffusion processing involved longitudinal sectioning of the annealed couple via precision wire electrical discharge machining, followed by sequential polishing using colloidal silica suspension. The microstructural features of phases and grains in the diffusion couple were characterized using a TESCAN S8000 GMH ultra-high-resolution scanning electron microscope (SEM, TESCAN, BrNo, Czech) equipped with an Oxford Instruments Nordlys Electron Backscatter Diffraction (EBSD, Oxford Instruments, Oxford, UK) detector. EBSD mapping and backscattered imaging were performed at an acceleration voltage of 20 kV and a current of 20 nA. Based on the inherent characteristics of the alloy, the scanning step size was set to 1.4–2.5 μm. The EBSD data were analyzed with Channel 5 software (Version 5.12.74.0). The crystal structure of pure Mg served as the reference pattern for the HCP phase, while the MgSc crystal structure was selected as the calibration standard for the BCC phase. Local chemical compositions were quantitatively point-to-point mapped via energy-dispersive X-ray spectroscopy (EDS, Oxford Instruments, Oxford, UK) with the same acceleration voltage on SEM. The mapping direction was along the diffusion direction. 

### 2.2. Nano-Indentation Creep Deformation and Continuous Stiffness Tests

The creep resistance evaluation was performed using Nano Indenter G200 (KLA Instruments, Phoenix, AZ, USA). We tested 20 indentation points stepwise at 20 different positions with different Sc compositions. These 20 indentation points were arranged in 2 rows parallel to the diffusion direction (composition gradient of Sc) to minimize interference from surface defects and grain boundaries. A spacing of 50 μm was maintained between adjacent indentation positions to avoid elastoplastic interactions. Based on the reference nano-indentation parameters for other Mg diffusion couples [12,47] and pre-test parameter tuning, a load-controlled mode was employed with a peak load of 10 mN (which can achieve a sufficient plastic zone depth to probe bulk-like creep behavior) applied at a constant loading rate of 1 mN/s. The maximum load was held for 100 s to ensure steady-state creep establishment. Subsequently, the specimen was unloaded with a 75 s thermal drift correction hold segment at 90% of the peak load during the unloading.

For strain rate sensitivity characterization, four microregions with different Sc compositions were probed via the continuous stiffness measurement (CSM) methodology to acquire hardness–depth profiles under varying strain rates (0.01 s^−1^, 0.05 s^−1^, and 0.1 s^−1^). A constant penetration depth of 1000 nm was maintained for all tests. The stabilized hardness values were determined by averaging data from the region (400–1000 nm depth) to minimize surface effects.

After a nano-indentation test, the chemical composition of Sc near the indentation was detected using low-voltage EDS point mapping. And transmission electron microscopy (TEM) observation was performed including bright-field (BF) imaging and selected area electron diffraction (SAED). The focused ion beam (FIB) was applied to prepare TEM specimens from two different near-indentation regions, but with significant differences in chemical composition. The ordering/disordering transition in BCC phases was addressed via TEM using a Talos F200X microscope (Thermo Fisher Scientific, BrNo, Czech) at an accelerating voltage of 200kV.

### 2.3. Data Analyses of Nano-Indentation Creep Deformation Test

The creep response of nano-indentation can be quantitatively described by the stress and time during the load holding stage, that is, following the Norton power-law model [48].

(1) $$\sigma =\frac{P}{S}$$

Among them, *P* represents the instantaneous load, and *S* represents the instantaneous contact area between the indenter and the sample during the indentation process.

(2) $$\dot{\varepsilon }=A\sigma^{n}\exp\left(-\frac{Q}{RT}\right)$$

In Equation (2), $\dot{\varepsilon }$ is the strain rate during holding, *σ* is the instantaneous stress, *A* is a constant, *n* is the stress exponent, *Q* is the creep activation energy, *R* is the universal gas constant (8.314 J·mol^−1^·K^−1^), and *T* is the temperature in Kelvin. Creep activation energy, *Q*, reflects the minimum energy required for atoms to cross the potential barrier during creep. Its value is usually related to the diffusion mechanism and characterizes the energy barrier required for atoms to migrate from high-energy positions to low-energy positions. In this study, all nano-indentation creep tests were conducted at room temperature (298 K). Thus, *T* = 298 K was used in the analysis.

Meanwhile, the strain rate $\dot{\varepsilon }$ can also be expressed by indentation depth, as in Equation (3).

(3) $$\dot{\varepsilon }=\frac{1}{h}\frac{dh}{dt}$$

In this nano-indentation experiment, the Bercovich triangular pyramid indenter was used. Therefore, under ideal conditions, the relationship between its contact area and indentation depth is shown in Equation (4).

(4) $$S=24.56h^{2}$$

When substituting Equations (1), (3) and (4) into (2), integrating the stress *σ* and time *t*, and then taking the logarithm, the relationship between creep stress and creep time during the indentation holding process can be obtained as shown in Equation (5).

(5) −nln(σ)=ln(t)+lnB−Q/RT

where *B* is constant and *ln*(*σ*) and *ln*(*t*) show a linear relationship. The slope of the linear regression plot is associated with the negative reciprocal of the creep stress exponent *n*.

### 2.4. Data Analyses of Nano-Indentation Continuous Stiffness Measurement

The strain rate sensitivity response of nano-indentation is derived. The material hardness *H* measured by the nano-indentation can be expressed as

(6) $$H=C{\dot{\varepsilon }}^{m}$$

where *C* is a constant, $\dot{\varepsilon }$ is the indentation strain rate ($\dot{h}/h$), which can be expressed as the loading rate divided by the load value, and *m* is the strain rate sensitive factor, whose value is always greater than and close to 0. Take the logarithm of both sides of Equation (6),

(7) $$\ln(H)=m\cdot \ln(\dot{\varepsilon })+\ln(C)$$

In this way, the linear relationship between the logarithm of hardness and the logarithm of strain rate can be obtained, and its slope is the strain rate sensitive factor *m*.

(8) $$m=\frac{d\ln(H)}{d\ln(\dot{\varepsilon })}$$

## 3. Results and Discussion

### 3.1. Microstructural Characterization

Figure 1a schematically shows, and the phase map in Figure 1c experimental evidences, the HCP/BCC/HCP sandwich structure of the diffusion couple after annealing at 630 °C (903 K) for 72 hours, which contains three phases. On the left is the end-member of the Mg-rich HCP phase, and on the right is the end-member of the Sc-rich HCP phase. Both HCP phases exist before annealing. In between, a layer of the BCC phase has been generated.

Figure 1b shows the EBSD orientation mapping projected by the colors of the inverse pole figure (IPF). The Mg-rich HCP phase (on the left, in green) and the Sc-rich HCP phase (on the right, in pink) show extremely coarsened grains. There are no grain boundaries in the figure horizon. The BCC phase exhibits an increasing grain size from the original weld interface towards the left and right sides, as the initial weld interface can serve as a preferential heterogeneous nucleation site. However, the initial weld interface was generally smeared out during annealing diffusion. Thus, the vicinity of the original weld interface produces fine equiaxed grains, and a progressive coarsening trend emerges with increasing distance from the interface, accompanied by preferential grain elongation parallel to the diffusion direction. Due to the effect of heterogeneous nucleation on the initial welding interface, which is dominant in grain size distribution, it is not proof-positive that the grain size in the BCC region is related to the varying Sc contents.

Figure 1b also exhibits the EDS-detected chemical composition–distance profiles. The profile of Sc is in yellow, and Mg is in black. Thanks to the reaction diffusion between Mg and Sc, not only is the BCC phase generated but also the continuous chemical composition gradients are formed in individual phases, especially in the BCC layer. There is a discontinuous composition transition taking place at the Mg-rich HCP/BCC interface, where the Sc composition abruptly increases from 11.66 at.% to 22.78 at.%, indicating the solid solubility of 630 °C (903 K) on the phase diagram. Figure 1b also schematically shows the direction of EDS scanning, which is parallel to the composition gradient of Sc. Thus, the nano-indentations are implemented along the same direction, such that the composition dependence of creep deformation behaviors can be figured out.

Figure 1c shows a backscattered SEM image of the diffusion couple, together with the phase identification image in the inset and the marked interfaces. The SEM image evidences a well-developed surface without cracks and the well-developed phase boundaries between HCPs and BCC. Although the grain size morphology changes with diffusion distance, there is neither cracking nor are there discontinuous defects inside the BCC phase. We can identify the trace of the initial weld interface only by SEM observation, indicating a high quality of diffusion bonding to prepare the diffusion couple. The generated phase interfaces of Mg-rich HCP/BCC and BCC/Sc-rich HCP are almost perpendicular to the composition gradient, indicating that a planar diffusion has taken place in the diffusion couple, such that the chemical compositions perpendicular to the Mg/Sc inter-diffusion are almost the same [49,50], and the nano-indentation perpendicular to the phase interface is along the chemical composition gradient of Sc.

Considering the experimental results showing that (1) the BCC layer with a single-phase thickness ≈ 650 μm is enough to implement dozens of nano-indentations with the necessary spacing, (2) there is a big enough grain size to avoid the effect of a grain boundary, (3) there is a composition range of Sc from ~23% to ~40% with an appropriate (not too steep) spatial gradient, (4) there is a high metallurgical quality of the BCC phase without cracking and discontinuous defects, the prepared BCC layer is qualified to implement a nano-indentation test inside the individual grains and then further to explore the composition dependence of creep behaviors of Mg-Sc BCC alloys at room temperature.

### 3.2. Nano-Indentation Creep Behavior

**Figure 2:**
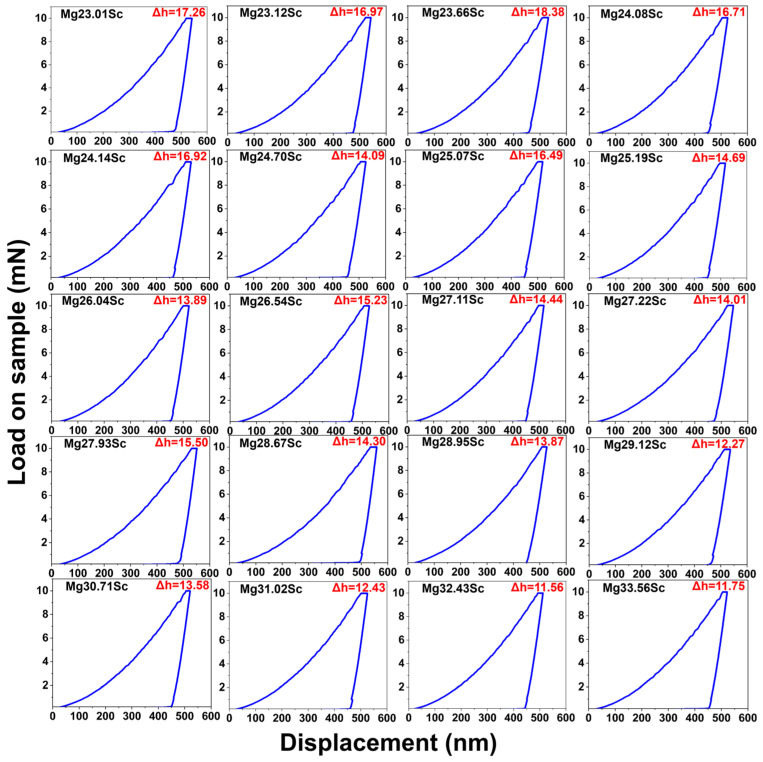
Load–displacement curves obtained through nano-indentation testing on BCC-structured microregions with varying Sc compositions ranging from 23.01 to 33.56 at.%.

Figure 2 displays the load–displacement responses obtained by nano-indentation testing on BCC-structured microregions with varying Sc compositions from 23.01 to 33.56 at.% under 10 mN maximum load. The selection of this target composition range and the corresponding area on the diffusion couple was considered to ensure the appropriate composition gradient to track the continuous variation in creep behaviors with Sc contents, and the spacing of 50 μm between two individual nano-indentations was set to avoid elastoplastic interreaction. However, the region with 35 at.%~60 at.% Sc of the BCC phase was dropped in this work. The chemical composition gradient of Sc was too steep to maintain the high spatial resolution of the chemical composition and the uninterrupted creep properties (see Figure 1b). Furthermore, in the Sc content lower than 23 at.%, the HCP phase can be detected in the diffusion couple. There are plateaus during the 100 s dwell period in all the tested samples (see the holding-stage characteristics in individual insets) demonstrating the occurrence of creep deformation. The creep displacement Δ*h* (defined as depth variation during load holding) fluctuates and decreases from 17.26 nm to 11.75 nm as the Sc content increases from 23.01 at.% to 33.56 at.%. It is noteworthy that the nano-indentation penetration depth is measured at ~500nm, as shown in Figure 2. The size of the elastoplastic affected zone introduced by the indenter is assumed to be 5–10 times the penetration depth [51,52,53], with the maximum size less than 5 μm. In comparison with the BCC grain size in Figure 1, the dislocation movement in the elastoplastic affected zone will hardly be blocked by the grain boundary. The variation in creep displacement is primarily attributed to Sc alloying.

There is an empirical formula in conventional tensile/compression experiments to describe creep displacement variation with time.

(9) $$u_{c}=u_{0}+a{\left(t-t_{0}\right)}^{b}+kt$$

where *u_c_* is the creep displacement, *u*_0_ is the displacement at the beginning of the test, *t* is time, *t*_0_ is the start time of creep, and *a*, *b*, and *k* are the fitting parameters. In a nano-indentation creep experiment, the loading–unloading curve is not ‘smooth’ (not differentiable everywhere). Since we only focus on the holding stage for creep, the start time and displacement of creep should be extracted from individual nano-indentation curves, and then be subjected to numerical fitting and renormalized to zero. By renormalization, Equation (9) is simplified into

(10) $$h_{c}=at^{b}+kt$$

where *h_c_* is the nano-indentation creep depth and *u*_0_ and *t*_0_ will be normalized to zero.

**Figure 3:**
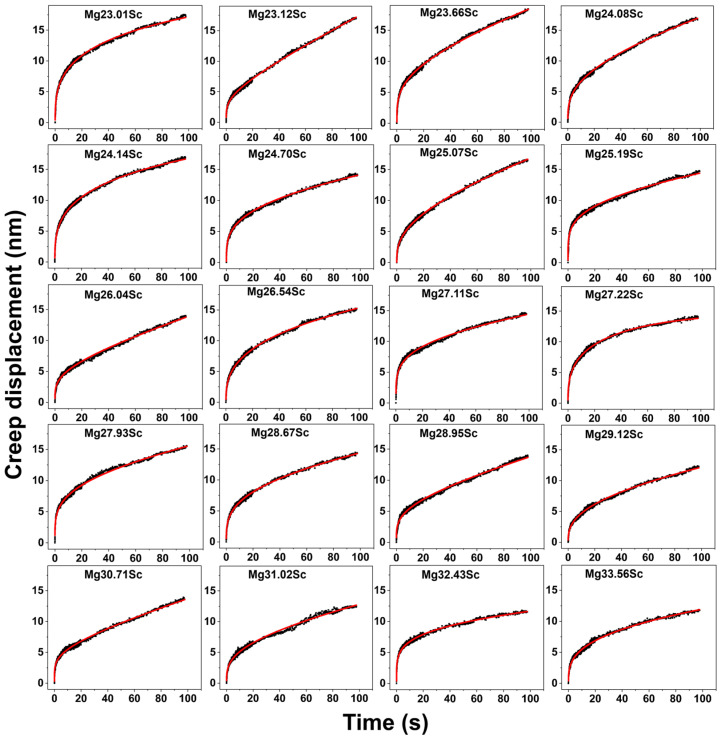
Creep displacement–time curves and fitted curves for BCC-structured MgSc alloys with different compositions.

Based on Equation (10), a power function regression analysis was conducted on the creep displacement–time relationship. In Figure 3, the experimental displacement–time curves are compared with the fitting curves using Equation (10). The experimental curves were obtained under different composition conditions after zero-point correction of the displacement–time data, holding the load for 100 s. All the experimental curves show the features of creep deformation, i.e., the deformation can be divided into two stages: an initial transient creep and subsequent steady-state creep. In the initial stage, the displacement shows a rapid increase with time. Subsequently, the deformation rate gradually slows down, and the creep curves decay. After entering the stable stage, the displacement turns to a linear increase. These behaviors are well described by Equation (10). The fitted curves and the experimental curves show good consistency with the fitted correlation coefficient R^2^ values higher than 0.98. The specific parameters (the fitting constants of *a*, *b*, and *k* and the corresponding R^2^ values) are detailed in Table 1. Fitted parameters *a*, *b*, and *k* and correlation coefficients R^2^ were obtained by fitting creep displacement–time curves for different Sc contents using Equation (10).

**Figure 4:**
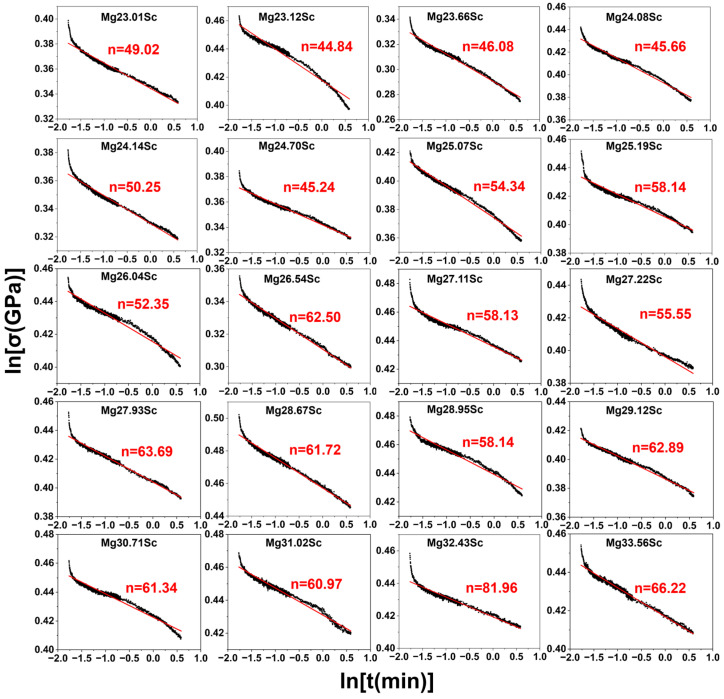
ln(*σ*)-ln(*t*) relationship curves and creep stress exponent (*n*) for BCC-structured MgSc alloys with different compositions.

Figure 4 depicts the creep stress exponent (*n*) with the composition of Sc, which was derived using Equation (5). The calculated n values increase from 49.02 to 66.22 as Sc% rises from 23.01 at.% to 33.56 at.%. The enhancement of *n* correlates with improved creep resistance, since it indicates a greater inherent resistance to time-dependent plastic flow under constant load conditions.

**Figure 5:**
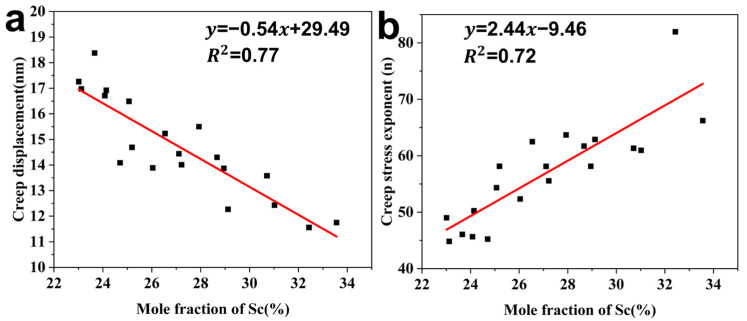
The variations in (**a**) creep displacement (Δ*h*) and (**b**) creep stress exponent (*n*) with Sc content.

Figure 5 exhibits a good linear Sc composition dependence of creep displacement and the stress exponent. Considering the random error sensitivity of nano-indentation [54], the small error fluctuations among individual tests and the good performances of linear regressions indicate the repeatability and reliability of the experimental results. Figure 5a quantifies the variation in creep displacement (Δ*h*) under a 10 mN holding load with Sc contents. It also describes the composition-dependent creep resistance of BCC-structured MgSc alloys. The experimental Δ*h* exhibits an inverse and linearly proportional relation to the Sc content, yielding a slope of −0.54 nm/at.% and R^2^ = 0.77. This trend quantitatively confirms progressively inhibited creep activity with Sc enrichment in the BCC phase.

Figure 5b reveals a proportional increase in stress exponent (*n*) with Sc content, following a linear dependence of the slope: 2.44 and R^2^ = 0.72. The monotonic increase in *n* from 49.02 to 66.22 also demonstrates that the addition of Sc to the BCC matrix can effectively strengthen time-dependent deformation resistance. This correlation aligns with prior investigations [34] showing that Sc additions intensify elastic interactions between solute atoms and dislocations, thus enhancing the comprehensive mechanical properties of the alloy.

There is a specific connection between the stress exponent *n* and the creep deformation mechanism [55]. All *n* values varying with Sc contents are greater than 3 in this work, indicating that dislocation movement plays a significant role in creep behavior [56], and the grain boundary sliding and grain boundary diffusion creep mechanisms are not applicable under the current experimental conditions. As is mentioned in the previous paragraph, there are differences in magnitude between the size of indentation (elastoplastic affected zone) and the grain size in this work. The probability of the indentation arising mainly in the grain boundary area was low enough. Most of the indentations were implemented in the intragranular area. Therefore, the effect of grain boundaries on the creep deformation behaviors is less important in this work.

**Figure 6:**
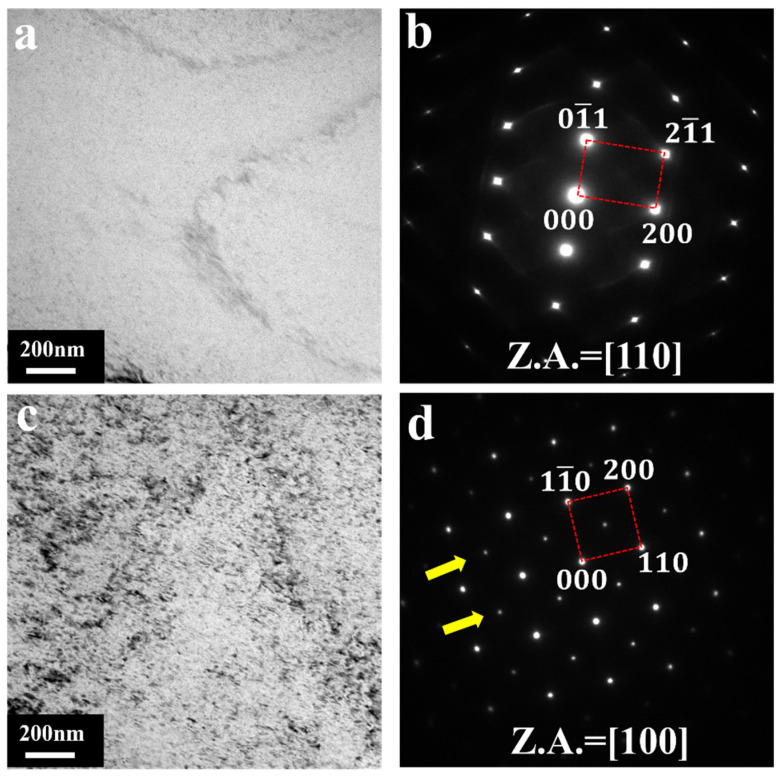
TEM test results of microregions. (**a**) BF image. (**b**) SAED at Mg 21.67 at.% Sc. (**c**) BF image. (**d**) SAED at Mg 32.66 at.% Sc. The yellow arrows highlight the additional reflection array at $\frac{1}{2}\left\{200\right\}$, indicating the formation of B2 ordered structure.

Figure 6a shows the BF image of the BCC-structured MgSc alloy at an Sc content of 21.67 at.%. The SAED pattern corresponding to Figure 6a is shown in Figure 6b, which proves that this region is a single disordered BCC phase. Figure 6c,d show the BF images and SAED at Mg 32.66 at.% Sc. It can be seen that the additional spots appear under the [100] zone axis and are located at 1/2{200}, indicated by yellow arrows. These additional spots suggest that the formation of the BCC-B2 ordered structure occurs at Mg 32.66 at.% Sc. This is also consistent with what was mentioned in previous studies: the BCC-B2 phase exists when the Sc content is within the range of 35–50 at.% Sc composition [16,25].

With the SAED patterns, we estimated the lattice parameter a = 3.581 Å at Sc = 21.67 at.% and a = 3.690 Å at Sc = 32.66 at.%. It is evidenced that the alloying of Sc results in BCC lattice expansion. The random solid solution of Sc leads to local lattice distortion, which hinders the movement of dislocations and enhances creep resistance. Moreover, the change in lattice parameter results in a varied magnitude of Burgers vector, then influences the pre-exponential factor A and flow stress in the equation for the creep rate (Equation (2)). On the other hand, with an increasing Sc content, there is atomic-scale ordering of B2 taking place in the disordered BCC matrix, as confirmed by SAED observations shown in Figure 6d. These ordered precipitates possess a significantly higher energy barrier for dislocation penetration compared to the disordered matrix, as predicted by theories of precipitate strengthening [57]. Consequently, dislocations interacting with these precipitates experience increased drag forces, necessitating thermally activated climb processes to bypass obstacles [58,59]. This climb-controlled bypass mechanism inherently reduces dislocation mobility, leading to the observed decrease in creep displacement and the significant enhancement in overall creep resistance of the alloys.

### 3.3. Nanometer Indentation Strain Rate Sensitivity

**Figure 7:**
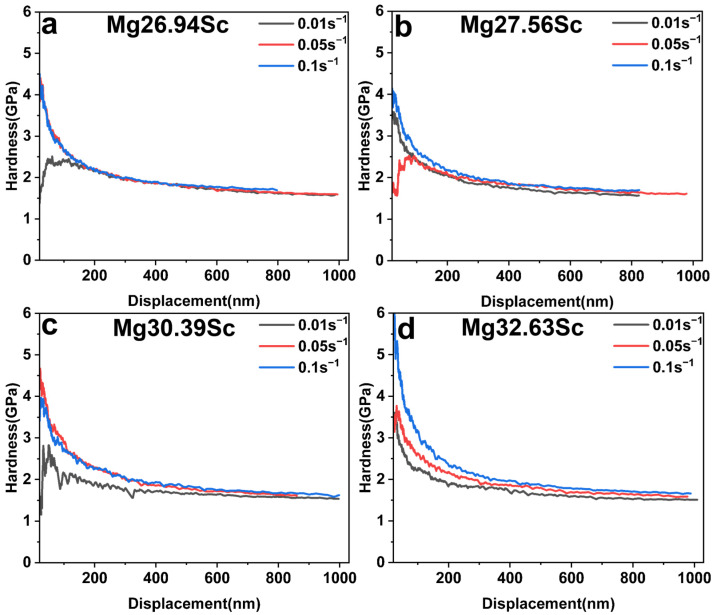
The hardness–depth curves of BCC-structured MgSc alloys with different compositions of Sc measured at various strain rates: (**a**) Mg 26.94 at.% Sc, (**b**) Mg 27.56 at.% Sc, (**c**) Mg 30.39 at.% Sc, (**d**) Mg 32.63 at.% Sc.

Figure 7 displays the hardness–depth profiles of the samples measured via the CSM method under various strain rates (0.01 s^−1^, 0.05 s^−1^, and 0.1 s^−1^). At shallow indentation depths (<400 nm), the hardness values exhibited significant fluctuations with increasing depth. However, the curves were gradually stabilized when the indentation depth exceeded 400 nm. Consequently, the average hardness values within the 400–1000 nm depth range were selected as the experimentally determined hardness. The results reveal an increasing trend in hardness with higher strain rates, indicating notable strain rate sensitivity of hardness in BCC-structured MgSc alloys. Furthermore, the influence of the strain rate on hardness becomes more pronounced with an increasing Sc content, as evidenced by the growing discrepancy between the curves. In terms of composition dependence, the stabilized hardness values remained approximately 2 GPa across all tested compositions, suggesting that Sc additions within this compositional range did not effectively enhance the hardness of Mg-Sc alloys.

**Figure 8:**
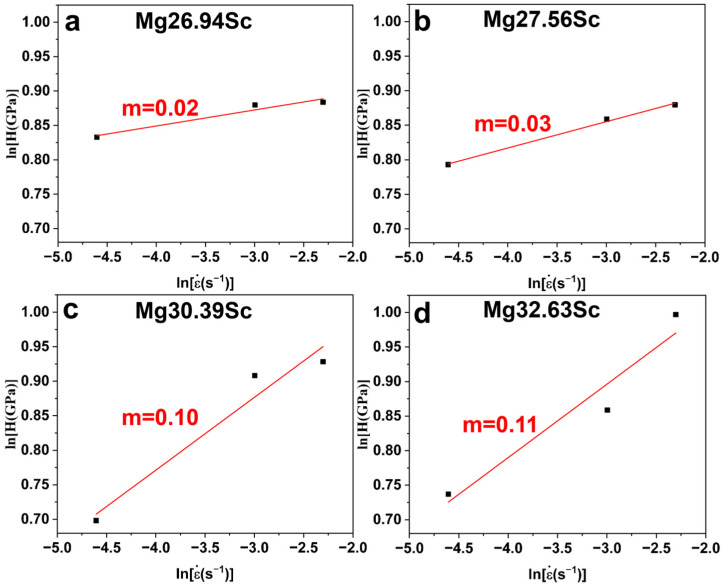
ln(*H*)-ln(*ε*) relationship curves and strain rate sensitivity index (*m*) of BCC-structured MgSc alloys with different compositions: (**a**) Mg 26.94 at.% Sc, (**b**) Mg 27.56 at.% Sc, (**c**) Mg 30.39 at.% Sc, (**d**) Mg 32.63 at.% Sc.

Figure 8 shows the strain rate sensitivity index (*m*), as evaluated by the ln (*H*)-ln ($\dot{\varepsilon }$) relationship with Equation (8). With the increase in Sc content from 26.94 at.% to 32.63 at.%, the *m* value increases from 0.02 to 0.11. The creep stress exponent (*n*) ascertained by nano-indentation creep and the strain rate sensitivity index (*m*) ascertained by CSM were obtained individually. The values of *m* and *n* from individual tests approximately satisfy the theoretical constraint of *m* = 1/*n*, also indicating the reliability of the results.

The present authors have studied the correlation between solvent diffusion and creep properties [12] and measured the diffusion coefficients in BCC-structured Mg-Sc alloys [45,46]. Sc acts as a diffusion-slowing solute in BCC Mg, leading to a decrease in the solvent diffusion of Mg atoms in Mg-Sc BCC solid solution, which may impose additional drag or friction on the movement of dislocations when the content of Sc is increased. More importantly, the enhanced *m* with the Sc content is not linear, showing a sudden increase at around 30 at.% Sc. According to the Mg-Sc binary-phase diagram, this may correlate with the formation of the chemically ordered B2 precipitates demonstrated in Figure 6. These ordered B2 precipitates act as localized barriers and obstacles to dislocation motion [60]. The effect amplifies as the strain rate increases.

## 4. Limitations

While this study provides valuable insights into the Sc composition dependence of creep behavior in bcc Mg-Sc alloys, several limitations warrant acknowledgment:

1. Only the material intrinsic creep resistance, the stress exponent, and the intrinsic strain rate sensitivity at room temperature, which are purely influenced by the chemical composition of Sc, have been studied. The creep deformation behaviors at elevated temperatures, which are activated by thermal physics, and the mechanisms of grain boundary sliding and grain boundary diffusion have not been considered yet. They are left for future works.
2. Strain rate range constraint: The nano-indentation creep tests were conducted within a defined strain rate range of 0.01 s^−1^ to 0.1 s^−1^. While appropriate for probing local creep resistance, this relatively narrow window may restrict the comprehensive quantification of the strain rate sensitivity exponent (m), potentially limiting the resolution of its composition-dependent evolution.
3. Microstructural correlation gap: The conclusions regarding strengthening mechanisms are based solely on mechanical property measurements (n, m). The absence of complementary microstructural characterization (e.g., TEM for dislocation substructures or precipitate analysis) prevents a definitive mechanistic interpretation of the observed composition–property relationships. Future work integrating microstructural probes is essential to elucidate the underlying mechanisms driving the enhanced creep resistance.

## 5. Conclusions

The composition-dependent creep resistance and strain rate sensitivity of BCC-structured Mg-Sc alloys were investigated through a nano-indentation creep deformation test and continuous stiffness measurement on the Mg-Sc binary diffusion couple. The key findings are summarized as follows:

1. Sc significantly improves the room-temperature creep resistance of BCC-structured MgSc alloys. The creep displacement Δ*h* decreases from 17.26 nm to 11.75 nm, while the stress exponent *n* increases from 49.02 to 66.22 as the Sc content rises from 23.01 at.% to 33.56 at.%. The composition variations in creep displacement and creep stress exponent indicate a better creep resistance of a Mg-Sc binary alloy at high-Sc compositions in comparison with low-Sc compositions. TEM characterization reveals that this enhancement originates from Sc-induced ordered structures, which act as practical barriers to dislocation motion through dynamic drag effects during creep deformation.
2. While the stabilized hardness remains at approximately 2 GPa across all compositions, the strain rate sensitivity index *m* increases from 0.02 to 0.11 with a higher Sc content at room temperature. This phenomenon is attributed to the proliferation of short-range obstacles (e.g., ordered structure) that amplify the activation barriers for dislocation glide under strain rate variations.

## Figures and Tables

**Table 1:** Fitted parameters *a*, *b*, *k* and correlation coefficients R^2^ obtained by fitting creep displacement–time curves for different Sc contents using Equation (10).

No.	Sc at.%	a	b	k	R^2^
1	23.01	4.3149	0.3175	−0.0147	0.9946
2	23.12	3.4316	0.2821	0.0158	0.9935
3	23.66	1.9768	0.3581	0.0189	0.9932
4	24.08	2.6334	0.2431	0.0586	0.9923
5	24.14	3.0238	0.3142	0.0428	0.9937
6	24.70	4.4832	0.2188	0.0221	0.9810
7	25.07	2.4008	0.3014	0.0320	0.9903
8	25.19	2.7927	0.2506	0.0495	0.9884
9	26.04	3.0589	0.2039	0.0589	0.9902
10	26.54	4.1954	0.2437	0.0271	0.9926
11	27.11	4.5016	0.2236	0.0183	0.9843
12	27.22	4.1373	0.3185	−0.0115	0.9959
13	27.93	2.9581	0.3583	−0.0067	0.9953
14	28.67	3.7173	0.2733	0.0547	0.9945
15	28.95	3.6620	0.3489	−0.0441	0.9937
16	29.12	2.4741	0.3439	0.0481	0.9969
17	30.71	4.0704	0.2161	0.0065	0.9854
18	31.02	2.6011	0.2266	0.1001	0.9959
19	32.43	3.0927	0.3041	0.0183	0.9941
20	33.56	2.5880	0.3196	0.0068	0.9901

## Data Availability

The data presented in this study are openly available in Mendeley Data at 10.17632/yr69v4x52k.1.
